# First Report of *Uncinaria hamiltoni* in Orphan Eastern Mediterranean Monk Seal Pups in Greece and Its Clinical Significance

**DOI:** 10.3390/pathogens10121581

**Published:** 2021-12-03

**Authors:** Anastasia Th. Komnenou, George A. Gkafas, Evangelia Kofidou, Joanne Sarantopoulou, Athanasios Exadactylos, Eleni Tounta, Kimon Koemtzopoulos, Panagiotis Dendrinos, Alexandros A. Karamanlidis, Frances Gulland, Elias Papadopoulos

**Affiliations:** 1 School of Veterinary Medicine, Faculty of Health Sciences, Aristotle University of Thessaloniki, 54627 Thessaloniki, Greece; natakomn@vet.auth.gr (A.T.K.); evikofidou@gmail.com (E.K.); 2 MOm/Hellenic Society for the Study and Protection of the Monk Seal, 10682 Athens, Greece; e.tounta@mom.gr (E.T.); k.koemtzopoulos@mom.gr (K.K.); p.dendrinos@mom.gr (P.D.); akaramanlidis@gmail.com (A.A.K.); 3 Department of Ichthyology and Aquatic Environment, School of Agricultural Sciences, University of Thessaly, 38446 Volos, Greece; gkafas@uth.gr (G.A.G.); saradopo@uth.gr (J.S.); exadact@uth.gr (A.E.); 4 Karen C. Drayer Wildlife Heath Center, School of Veterinary Medicine, University of California, Davis, CA 95616, USA; francesgulland@gmail.com; 5 Marine Mammal Commission, Bethesda, MD 20814, USA

**Keywords:** Mediterranean monk seal, *Uncinaria hamiltoni*, anaemia, morbidity, mortality, COI, rDNA

## Abstract

The Mediterranean monk seal (*Monachus monachus*) is classified by the IUCN as “endangered,” with a global population estimated to number fewer than 800 individuals. Our understanding of the biology and health status of the species is still limited, rendering every medical case a challenge for conservationists and veterinary clinicians. Although studying and managing disease in wild marine hosts is complex and challenging, studying and mitigating the effects of any disease to the Mediterranean monk seal is of utmost importance for conservation. The aim of this study was to document for the first time the presence of the hookworm *Uncinaria hamiltoni* in rehabilitated Mediterranean monk seal pups in Greece. A detailed examination protocol was followed for all pups that live-stranded over 30 years in 22 different locations, including physical, parasitological, and other examinations. Hookworms (adults and/or eggs) were detected in all the fecal samples, from all animals. Molecular identification using MtDNA (COI) and ribosomal DNA (D2/D3 28S and internal transcribed spacer [ITS] regions) identified the nematode species as *Uncinaria hamiltoni*. The clinical impacts and the benefits of anthelmintic treatment as a tool for the conservation management of the species are discussed.

## 1. Introduction

The Mediterranean monk seal (*Monachus monachus*) is the most endangered pinniped in the world and a flagship species for marine conservation. Following centuries of human persecution and habitat loss, the species has been extirpated from most of its historical range and is currently classified as “endangered” by the International Union for the Conservation of Nature (IUCN) [1,2]. Mediterranean monk seals survive in three small, isolated subpopulations: two in the northeastern Atlantic (i.e., Cabo Blanco and archipelago of Madeira) and one in the Eastern Mediterranean Sea [3]. The latter subpopulation currently occupies more than 90% of the species’ range, is estimated to number approximately 400 individuals, and has been a focal point of systematic research and conservation efforts since the early 1990s. Mediterranean monk seals in the Eastern Mediterranean are currently showing encouraging signs of population recovery. This recovery is evident in the re-establishment of Mediterranean monk seal populations in areas where they had become extinct [4] and in the presence of monk seals in closer proximity than previously to humans and their activities [5]. The latter fact increases the risk for zoonoses, which can negatively affect both humans [6] and endangered species, such as the Mediterranean monk seal [7].

Parasites have coevolved for a long time with their hosts as part of the external environment [8,9,10] and may have a serious effects on biodiversity by influencing the behavior of individual hosts [11], by regulating the size of host populations [12], and by acting as ecosystem engineers [13]. Even though the consequences of parasitic infections are often not too serious and can provide lifelong benefits, in some cases detrimental effects could be caused for their hosts and clinical and subclinical diseases may develop [8,10,14]. Specific factors can compromise the parasite–host balance, increasing the occurrence of opportunistic diseases in the marine environment through shifts in the distribution of either hosts or pathogens [15]. These may include climate change and climate-mediated stress, behavioral traits, host sociality, population density, diet, habitat, age, sex, host immunocompetence, supplementary feeding, and animal translocations [16,17,18,19,20,21,22].

Parasites are important components of ecosystems [23,24,25]. Their impact on the conservation of an endangered species may be significant, as permanent or temporal declines of host populations can result from parasitic diseases [26,27,28]. Mediterranean monk seals are potentially vulnerable to all major groups of pathogens and parasites, yet there are few available data on the health threats to this endangered species. Understanding the health issues (potentially) affecting the Mediterranean monk seal, especially parasitic infections and the factors influencing the epidemiology of disease, is essential for determining the impact of parasites on population health and developing effective conservation measures for the species.

Several parasites have been identified in pinnipeds. Among them are hookworms of the genus *Uncinaria* (Frölich, 1789 Nematoda: Ancylostomatidae), which are intestinal hematophagous parasitic nematodes. Thirteen species have been recognized in carnivores (Carnivora). Hookworms are common parasites in the otariids (eared seals, fur seals, and sea lions), rare in phocids (earless seals and southern elephant seals), and unreported in odobenids (walrus) [29]. One parasite, a hookworm of the genus *Uncinaria*, has been reported from the Mediterranean monk seal, but its identity and its clinical impact have not been assessed [30].

The aim of this study was to document for the first time the hookworm *Uncinaria hamiltoni* in monk seal pups rehabilitated in Greece, to describe the molecular systematics and morphology of the parasite, to discuss its clinical impact, and to evaluate anthelmintic treatment as a tool for the conservation of the Mediterranean monk seal.

## 2. Results

### 2.1. Clinical Evaluation

Thirty-one Mediterranean monk seal pups were stranded between 1990 and 2020. The detailed clinical examination on presentation revealed similar clinical signs in all animals, including severe weakness, dehydration, malnutrition, mild to severe skin lesions, oral mucosal pallor, dyspnea, and ocular and nasal mucous or mucopurulent discharge. Age determination, length measurements, and weight evaluation of the animals that survived and were released, as well as the ones that died, are presented below. A summary of arrival and release/death age, length, and weight is presented in Table 1.

### 2.2. Body Condition and Body Mass Evaluation

Upon presentation, body condition was evaluated based on visual determination and was classified as poor, fair, and good. Upon arrival, stranded pups had an overall mean “body mass index” of 0.154 (n = 31, range 0.100–0.253) and an overall percentage difference from the expected “healthy body mass index” (n = 31, mean = 0.183, range 0.162–0.257) of −15.6% (n = 31, range −51.6%–26.1%). Five out of thirty-one pups (16.1%) had a critical body condition, eleven (35.5%) had a poor body condition, and fifteen (48.4%) were considered to have a good/fair body condition.

Pups that survived arrived with a “mean body mass index” of 0.160 (*n* = 16, range 0.111–0.253) and an overall percentage difference from the expected “healthy body mass index” of −14.1% (n = 16, range −34.5%–15.2%). Upon release, all animals (*n* = 16) had achieved a “healthy body mass index.” The “mean body mass index” of released animals was 0.405 (*n* = 16, range 0.345–0.456), with an overall percentage difference from the minimum “healthy body mass index” of 29.0% (*n* = 16, range 1.4%–47.4%).

Pups that died arrived with a “mean body mass index” of 0.148 (*n* = 15, range 0.100–0.225), with an overall percentage difference from the expected “healthy body mass index” of −17.0% (*n* = 15, range −51.6%–26.1%). All animals that died during rehabilitation did so either with the same “body mass index” as they were admitted or with a deteriorated one. The “mean body mass index” of animals at the time of their death was 0.149 (*n* = 15, range 0.141–0.225), and the overall percentage difference from the minimum “healthy body mass index” was −22.6% (*n* = 15, range −53.2%–25.3%) (Table 1). Neither arrival age nor arrival weight showed significant differences between animals that survived and died.

### 2.3. Hematology and Biochemistry

The findings of the hematological and biochemical examinations from the blood samples taken at the time of arrival of all monk seal pups are presented in Table 2.

### 2.4. Parasitological-Coprological Examination

**Figure 1:**
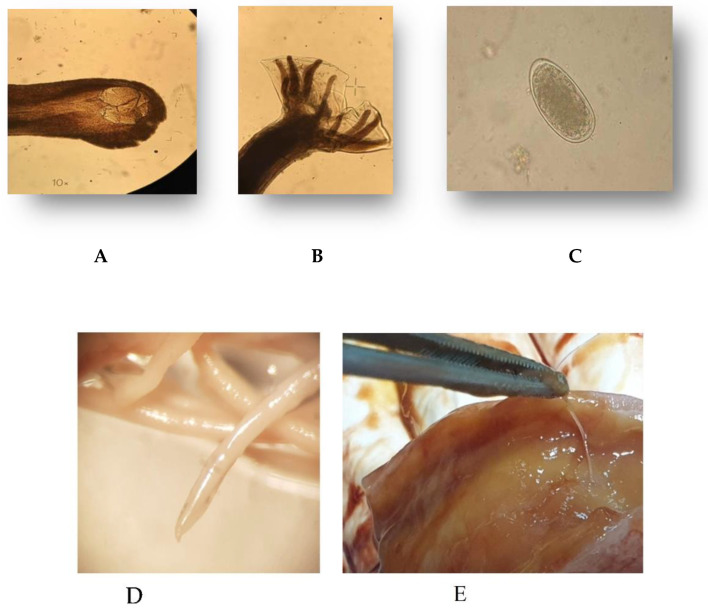
Adult *Uncinaria hamiltoni*, cephalic region with buccal capsule (**A**), bursa of the male (**B**), egg (**C**), adult rear end of female worm (**D**) and nematodes recovery from the intestinal tract (**E**).

The parasitological examinations of the rectal feces of all animals revealed nematode eggs, which were identified as *Uncinaria hamiltoni*, providing a prevalence of infection of 100%. In nine individuals, adult hookworms were also recovered. The mean and standard deviation of the dimension of the nematode eggs were 75 ± 0.18 × 45 ± 0.13 μm. The adult worms had the characteristic hookworm large funnel-shaped buccal capsule, with plates and teeth. The males had a large bursa with two separate lobes including the slender spicules. The body length of the parasites collected from the necropsied pups was calculated based on a sample of 25 female and 25 male nematodes. More precisely, the mean, ± standard deviation, and the range of the body length for female nematodes were 23.03 mm, ± 1.02, and 20.2–24.2, respectively, while for the male nematodes it was 14.9 ± 0.91, and 13.5–16.3, respectively. The details of the cephalic end and the male bursa and eggs are presented in Figure 1A,B.

The number of hookworm eggs in the fecal samples of each monk seal pup was correlated with its body condition and hematocrit (Packed Cell Volume, PCV). Pups in critical and poor condition and anemic (PCV 9.3%–28.6%) had significantly higher numbers of hookworms than the pups in good/fair condition (χ^2^ = 35.8, *p* < 0.0001). Higher hookworm infection intensity was associated with body condition and anemia (Table 3).

### 2.5. Molecular Identification

The aligned COI sequence revealed a length of 585 bp, while the rDNA regions revealed a length of 575 and 606 bp for the D2/D3 28S rDNA region and the amplified region that included the ITS rDNA subunits (partial 3′-end 18S, ITS-1, 5.8S subunit, ITS-2, and partial 5′- end 28S), respectively (Genebank accession numbers in Table 4).

BLAST results indicated that our specimen was 99.3% and 99.67% identical with *Uncinaria hamiltoni* for D2/D3 28S rDNA and ITSrDNA regions, respectively. The COI amplification suggests a novel sequence for *U. hamiltoni* (no other deposited COI region sequence was available). Comparing the COI sequence of our specimen with the sequences of the other three available species (*U. lucasi*, *U. lyonsi*, and *U. sanguinis*), *U. hamiltoni* differed from *U. lucasi* at 53 alignment positions (29 transitions and 24 transversions), from *U. lyonsi* at 51 positions (28 transitions and 23 transversions), and from *U. sanguinis* at 52 positions (29 transitions and 23 transversions). The disparity Index Test for Heterogeneity (bootstrap value 1000) among taxons revealed no significant departure from base frequency homogeneity after Bonferroni corrections.

**Figure 2:**
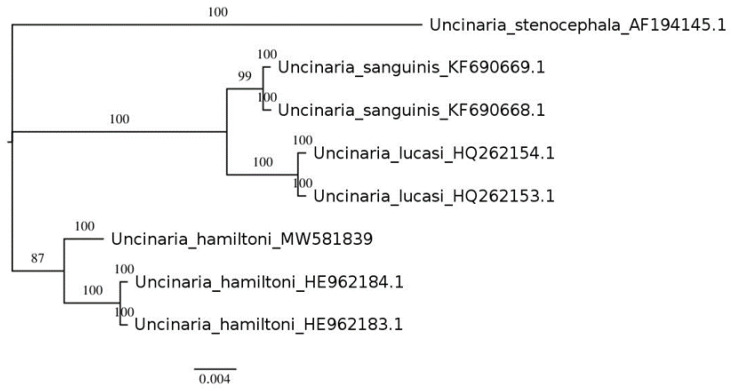
Phylogenetic tree for ITSrDNA region. Numbers shown next to the node are referred to Bayesian Inference posterior probabilities.

Among the available deposit sequences for all amplified regions, the phylogenetic tree (Figure 2) was conducted only for the ITSrDNA regions due to the maximum of the representative sequences. Supported clades (shape parameter for gamma distribution: 1.218) showed paraphyly within the *Uncinaria* species.

### 2.6. Treatment

Supportive treatment was initiated upon presentation with oral liquids and electrolytes (Almora Plus-ELPEN) and then continued by force feeding with a special homemade formula (fish porridge) and supplements; vitamin/mineral tablets (Aquavit ZVG LLP, Keighley, UK) iron; and extra vitamins B1, B6, and B12 (Neurobion tablets P&G Health Germany GmbH, Schwalbach am Taunus, Germany). In addition, all animals were treated with antiparasitic drugs, e.g., oral fenbendazole at a dose of 10 mg/kg b.w. for 3 days (Panacur tablets 250 mg/Intervet BV Hellas) or mebendazole 20 mg/kg b.w. for 2 days (Telmin TM tabl 500 mg Janssen Pharmaceuticals, Inc., Titusville, NJ, USA), depending on drug commercial availability and was repeated after 10 and 20 days along with antibiotics; oral amoxycillin + clavulanic 12 mg/kg b.w. (Synulox tablets 250 mg; Pfizer, Inc., New York, NY, USA); or metronidazole 10mg/kg b.w. (Flagyl tablets 400 mg Sanofi-Aventis, Paris, France), in order to prevent systemic inflammatory responses. Special care was given to skin lesions and ocular signs. The antiparasitic treatment was repeated after 10 and 20 days. Hematological, biochemical, and parasitological examinations were repeated every 15 days during the rehabilitation program. Successful treatment of parasites was confirmed with the absence of eggs after four repeated normal exams, along with the gradual restoration of hematology values (hematocrit) in sixteen (16) animals (eight males and eight females). After reaching a specific age (i.e., 5–6 months old) and weight 45–50 kg, these animals were safely released back into the wild. The remaining animals (15) died during the first days of rehabilitation due to severe anemia, hemorrhagic enteritis, bronchopneumonia, emaciation and starvation, and hypoglycemia (Table 5).

## 3. Discussion

### 3.1. Parasitic Species Identification

There is still a question of how many different hookworm species infect pinnipeds [29,34] based on morphological and morphometric characteristics of two species of pinniped hookworms that had been fully described previously: *Uncinaria lucasi* [35] and *Uncinaria hamiltoni* [36,37]. Further phylogenetic analyses in other specimens that had been isolated from nine different pinniped species showed seven independent evolutionary lineages or species, including the above-described species and five undescribed ones [30]. In eared seals (otariids), four *Uncinaria* species have been described [30,38]: *U. lucasi* in northern fur seals (*Callorhinus ursinus*) and a Steller sea lion (*Eumatopias jubatus*) [35,37]; *U. sanguinis* in Australian sea lions (*Neophoca cinerea*) [33,39,40,41,42]; *U. lyonsi* in California sea lions (*Zalophus californianus*) [43,44]; and *U. hamiltoni* in South American sea lions (*Otaria flavescens*) [36,45], South American fur seals (*Arctocephalus australis*), and in Australian fur seals (*Arctocephalus pusillus doriferus*). Molecular analyses conducted in other specimens suggested that they do not fit in the above descriptions; there are at least five undescribed *Uncinaria* species found in the California sea lion, the New Zealand sea lion, the Australian fur seal, the Mediterranean monk seal, and the southern elephant seal that still need to be described [30,38,46].

The taxonomic identity of the hookworms that parasitize the Mediterranean monk seal has not been reported. In our study, molecular evidence revealed that *Uncinaria* sequences were >99% identical to *Uncinaria hamiltoni* that are found in the Southern hemisphere (Cabo Polonio, Rocha, Uruguay [30]). The dispersal ability of the *Uncinaria hamiltoni* lineages follow a rather phylogenetically complex pattern, indicating a broader biogeographic pattern [37,44]. Our study revealed novel sequences of *U. hamiltoni* in the northern hemisphere, suggesting a cosmopolitan distribution of the parasite, and a dynamic evolutionary spatial pattern of the host–parasite interaction. However, other nematodes within the same genus (*Parafilaroides* spp.) have been described within pinnipeds [47] indicating an extended geographical distribution of helminths. The evolutionary outcome of dispersal is that parasite distribution may depend on the host’s dispersal dynamics [48] when dispersal is local (e.g., in large colonies as seen in pinnipeds). On the other hand, when dispersal is not local, parasites invest in an intermediate dispersal distance through selective pressure on the spatial dynamics of host–parasite interaction [49]. However, this co-evolutionary characteristic of dispersal may be due to environmental pressures, such as climate change or more direct anthropogenic impacts [50], or possibly due to species-level potential mechanics behind transmission and intermediate hosts [51].

### 3.2. Parasite Transmission

The life cycle of hookworms in most pinnipeds has not been fully clarified, but it is probably similar within otariid species [40,52,53]. *U. lucasi* is the only pinniped hookworm for which the life cycle has been experimentally completed in fur seals [54]. Contrary to other species, in otariids, *Uncinaria* spp. apparently do not migrate within the host to reach the intestine, and the lactogenic route is considered the main and probably the only form of transmission [40,52,55,56]. The free-living hookworm larvae infect the animals orally or percutaneously and then migrate through the tissues predominantly to the ventral abdominal blubber. Parasitic L3s are acquired by nursing pups from their mother’s milk through colostrum. Successful hookworm colonization of the intestine only occurs during the first week of the pup’s life. Then, the eggs are passed in the feces of the pup onto the rookery soil [53], hatched as free-living L3s, and then they penetrate the skin of seals or enter orally. Adult males are considered as dead-end hosts, as they cannot infect the pups [53] but could play a possible role in parasite dispersal.

The life cycle of hookworms in the Mediterranean monk seals is not known but might be similar to the one of other pinniped species. In other seal species that live in colonies, a sandy substrate is considered favorable for the development, survival, and transmission of free-living hookworm larvae [52,57]. Mediterranean monk seals in the Eastern Mediterranean Sea usually live in small family units and have shown to prefer marine caves often with a limited terrestrial surface covered with a sandy substrate [58], which can be favorable for the transmission of parasites. These factors, probably along with the fact that the lactation period in the Mediterranean monk seal is longer compared to other seals (up to 5 months), may contribute to an increased transmission rate. However, there are still some gaps in our understanding of the method of transmission, the pups’ age of eggs detection, and whether trans-mammary transmission plays a central role in infection. Further studies are required in order to elucidate these gaps, considering that in fur seal pups with hookworm infection, eggs cannot be detected in the feces over a certain age as hookworms fail to mature to produce ova [59].

### 3.3. Clinical Significance

Parasitic infections may be related to the development of clinical and subclinical diseases, causing several hematological and behavioral changes, reduced growth rates, and mortality in many cases [60,61]. Hookworm infections in humans, domestic animals, and wildlife species cause anemia due to chronic blood loss; tissue damage; significant inflammation in the mucosae and secondary bacterial infections; impairing digestion and absorption; retarded growth; and significant mortality in several wildlife species [38,62,63,64]. Two hookworms of the genus *Uncinaria* (*U. lucasi* and *U. hamiltoni*) are responsible for high mortality in young pinnipeds due to heavy parasitism of adult worms causing severe anemia and hemorrhagic enteritis [65,66,67] related to anticoagulant proteins the parasite secretes to the suckling site [66]. It is also suggested that hookworm infection in these species may increase susceptibility to other diseases, such as bacterial infection or trauma [68,69]. Anemia has been documented more commonly in canids, felids, and otariids and retarded growth only in otariids [38], along with severe mortality during the reproductive season (up to 70% of the total pups) [38,69,70]. The reasons for these high levels of mortality among eared seals are unknown. It seems that the parasite has developed a distinctive strategy and a special host–parasite relationship, resulting in remarkable adaptation in the marine lifestyle of the host. One of the reasons for the high pathogenicity in pinniped species could be the aggressive feeding behavior of hookworms to supply their high metabolic demands [69,71], making the animals experience the worst consequences such as anemia, retarded growth, and mortality (the highest levels of recorded in animal populations). Adult pinniped hookworms ‘live” fast and ‘die’ young, as they have little time to grow, mate, and release eggs (100% dead in 4–8 weeks). The relationship between hookworm infection intensity and clinical hookworm disease in pinnipeds is not entirely clear. It appears that the clinical appearance is directly related to the number of hookworms present; a single female hookworm has the ability to produce approximately 30,000 eggs a day [56,72]. The clinical impact of the hookworm infection on the health status of endangered Mediterranean monk seal pups has not been investigated or reported previously.

Even though hematological parameters and changes have been studied for pups of other pinniped species [73,74,75,76,77,78], few reports exist about the relationship of parasitosis on the hematological values of these pups and the implications for the assessment of their health status. In our cases, animals with a heavy parasitic burden had the more severe clinical pictures and consequences, as evaluated based on hematological and biochemical parameters. Although hematological analysis can be influenced by host specific differences and physiological stressors [79,80,81], it represents a valuable and acceptable non-invasive tool for the routine health screening of live orphan Mediterranean monk seal pups as well as the evaluation of the impact of a parasite to its host. Moreover, hookworm enteritis with secondary bacteremia, resulting in infections in multiple organs and cavities accompanied by anemia, has been observed in other pinniped species [54,65,69,82]. Anemia is quite common in young otariids primarily due to severe infestation and secondary to malnutrition. This complex of enteritis and anemia has been related to the high density of animals in the rookery, resulting in increased numbers of pathogenic parasites and bacteria in the environment, as pups can swallow sand resulting in a continual high pathogen burden [69].

### 3.4. Importance of Parasitic Burden

The body condition of pups, based on their “body mass index” at the time of their arrival, played an important role in their survival potential only in cases where it represented a critical body condition. All pups that arrived with a critical body condition died early during the rehabilitation process, while survival rates were more variable for pups arriving with a poor or good body condition. Overall, these data show that there might be other factors affecting the response of a pup to the treatment. One such factor could be the stage and intensity of hookworm infections. Studies support that the high mortality rates and the severity of hookworm-associated poor body condition has been related to the intensity of hookworm infection [56,83,84], whereas the comparatively low hookworm infection intensity has not been related to that [45,72,85]. In contrast, others support that pups found dead in better body condition demonstrated higher hookworm infection intensity compared to pups in poor body conditions [56,72,86], probably due to the fact that these pups were relatively younger and experiencing the effects of acute rather than chronic hookworm infection; therefore, adverse effects on body condition were not yet evident [38]. In our study the monk seal pups with high parasitic burden and low weight faced the worst of the consequences, since five of them had low PCV’s (9–18%) and critical body condition (>35% less). The association between high hookworm infection intensity and critical body condition in monk seal pups that died suggests that hookworm infection could have a serious impact on pup survival, apparently via nutrient and energy loss through gastrointestinal hemorrhage.

As there is a high association of hookworm infection with disease and mortality of pups in several pinniped species [56,87,88,89], undoubtedly the parasitic burden is one of the main factors that determine hookworm pathology and mortality. Our preliminary findings suggest that *Uncinaria hamiltoni* is similarly pathogenic in monk seals pups; hence, it is important to study the relationship of the hookworm and the host.

### 3.5. Treatment

Anthelmintic treatment for preventing or eliminating hookworm infection and improving pup survival in other seal pups has been investigated by using several compounds. Among them, disophenol sc showed variable effectiveness (<1–100%), and dichlorvos po showed high effectiveness (>99%), accompanied by side effects, e.g., diarrhea and toxicity in some pups [90,91,92]. Moreover, diethylcarbamazine, fenbendazole, levamisole, and morantel tartrate have also been used, but few details about their effectiveness are available [93] and cited in [53]. The most effective treatment was achieved with ivermectin sc at 200 μg/kg twice, 10 days apart (~96–100%), and fenbendazole at 10 mg/kg for 3 successive days [94,95]. In our cases, fenbendazole served orally at a dose of 10mg/kg for 3 days or mebendazole 20 mg/kg for 2 days, along with ampicillin, achieved successful treatment of parasites that was confirmed through the absence of eggs after four repeated normal exams along with the gradual restoration of hematology values (hematocrit). In addition, treated pups demonstrated significantly high growth rates after anthelminthic treatment. Iron and B12 complex were also effective for treating anemia. At this point, it should be stressed that the parasitological method used for counting eggs through stool examination suffers from limitations. Unfortunately, it was not applied in any of the standard quantitative coprological techniques, but instead it was used as the mean number of eggs per optical field, which does not fully correlate to the actual number of eggs per gram of feces. This approach was employed as we only wanted to compare any differences of the numbers of eggs during clinical treatment.

### 3.6. Parasite–Host Interaction

In our study, hookworms proved to be highly pathogenic, causing severe intestinal damage and anemia, which in a significant number of pups resulted in bacterial infection and death. Moreover, some seal pups had unusually high levels of parasites related to others. The reason this happened is somewhat unclear. It might be related with several factors, e.g., high density of seals in caves. Another possible explanation of the different levels of infestation might be subject to the differential genetic diversity among pups. Studies on Heterozygosity Fitness Correlations (HFCs) have shown that genomic diversity is often correlated with fitness-related traits due to genome-wide effects [48,96]. Furthermore, studies on marine mammals show the sex-specific impact of genomic diversity on pathogen load [97], which may be promoted by sexual selection.

In small populations, inbreeding is an unavoidable process. Studies in the literature show that heterozygous individuals are less likely to be infected by a range of parasites [97,98] and that correlations between homozygosity and susceptibility to parasite infections are often positive [48,96,98]. Population structure affects local effective population size, and thereby affects the level of inbreeding. Therefore, the considerable population structure observed in the monk seal [99,100] has the potential to influence the genetic impact on fitness to a greater extent than may be expected for other marine mammals (e.g., striped dolphin [101]). Moreover, local populations are more likely to be affected by short- term neutral forces (genetic drift). Therefore, a small effective population size can render local adaptation more difficult due to the stronger force of genetic drift compared to the selection force in small populations; consequently, a smaller effective population size may result in higher susceptibility to parasite infections.

The relationship between hosts and parasites represents a critical issue for wildlife conservation. The list of endangered species is increasing every day and especially for marine mammals, which are vulnerable to all the major groups of parasites. Studying the parasitic impact on the health status of animals is a critical step towards the implementation of appropriate management plans for their protection [25]. Therefore, in rehabilitation and breeding programs, parasitological surveys and treatments are essential for preventing the emergence of novel diseases and for the survival of the species [102].

## 4. Materials and Methods

Since 1990, 31 stranded monk seal pups (16 males and 15 females) had been rescued and rehabilitated in the Monk Seal Rehabilitation Centre of “MOm/Hellenic Society for the Study and Protection of the Monk seal” in cooperation with the School of Veterinary Medicine, Faculty of Health Sciences of the Aristotle University of Thessaloniki (AUTH) in Greece. Seal pups were found stranded in 22 different locations in Greece, from late August to mid-February. Upon presentation, age and weight estimation, morphometrics (length), body condition evaluation (body mass index), and detailed clinical examinations were performed; these examinations were repeated at specific intervals (every week for the first month and then every 15 days). After oral rehydration, blood samples were collected from the epidural vertebral vein by inserting a 18GA 2′ spinal needle between the dorsal spinous processes of the 3rd, 4th, or 5th lumbar vertebrae. The samples were transferred into SST and EDTA tubes for hematology and serum biochemical analysis. Fecal samples were collected for parasitological examinations and parasite genetic analysis, along with samples for microbiology and virology. In addition, thoracic and abdominal X-rays were performed in most cases. As a response to these diagnostic tests, an individually tailored medical treatment and rehabilitation program was established for up to 174 days.

### 4.1. Age Estimation

Pup age upon admittance was estimated from data related to observed births in the wild and rehabilitation data, i.e., using a combination of characteristics, namely the following: umbilicus presence, umbilicus state (open/closed), dentition stage, molting stage, weight, and length [103].

### 4.2. Weight Evaluation and Body Mass Index

Pup body condition was evaluated based on “body mass index,” which was estimated by dividing the weight (in kg) with the total length (in cm) (kg:cm ratio) at the time of their arrival and at the time of their death/release [89]. The “body mass index” of rehabilitated animals was compared to the one of healthy animals in the wild, which was found to be age dependent (MOm unpublished data). As measurements and age estimation of “healthy” animals of an intermediate age (20–120 days) in the wild were unavailable, the “healthy body mass index” was calculated by plotting the “body mass index” of wild (non-orphaned) young pups of a known age (2–5 days) along with the lowest “body mass index” of a released rehabilitated animal verified to have survived for at least 4 months following its release. The resulting trendline equation was used to calculate the minimum “healthy threshold body mass index” for all ages. The actual “body mass index” of every rehabilitated pup at its arrival and release/death age was compared to the “healthy” threshold index at the same age. The resulting values were calculated as percentage differences (+/−) between the actual “body mass index” and the “healthy body mass index.” These values (percentages) were then used to designate pups into the following categories: (1) good/fair body condition: x > −15%; (2) poor body condition: −15% < x <−35%; and (3) critical body condition: x < −35%.

### 4.3. Coprological Examination

A fresh fecal sample was collected directly from the rectum of each animal using a plastic glove and kept at 4 °C. All samples were examined in the Laboratory of Parasitology and Parasitic Diseases of the School of Veterinary Medicine of AUTH within 48 h of collection. Initially, each fecal sample was examined under a micro-stereoscope (Zeiss, Germany) to detect any worms expelled with feces. Worms that were found were collected, cleaned with saline, and placed into a vial with ethanol 90% for further morphological identification (for genus identification) using standard keys [104,105] and molecular analysis for species identification. The parasitological examination of the feces was carried out using a flotation method with zinc sulphate. The supernatant was examined under a microscope (Axioskope, Zeiss, Germany) in order to detect any parasitic elements.

### 4.4. DNA Extraction and PCR Amplification

Total genomic DNA was extracted from a pooled sample of specimens recovered from nine pups according to a phenol/chloroform-based protocol [106]. DNA was diluted in TE and stored in −20 °C. From the NCBI (GenBank^®^—www.ncbi.nlm.nih.gov/ (accessed on 30 September 2021) database, the only deposited genomic regions for identified *Uncinaria* species included one in MtDNA (Cytochrome oxidase subunit I (COI)-after) [107], one in ITS-rDNA, and two regions in 28SrDNA(D2/D3 and D18/D19), including the 18S 3′-end, ITS-1, and ITS-2, 5.8S subunit, and 28S 5′-end [34] (Table 2). PCR reactions were performed in 25 mL reaction mixtures containing ~ 10 ng template DNA, 5 mL of 10× PCR buffer (Invitrogen), 2.5 mM MgCl_2_ (Invitrogen, Waltham, MA, USA), 0.2 μL of 10 mM each deoxyribonucleotide triphosphate (dNTPs) (Invitrogen), 0.3 μL of each 10 mM primer (Operon-Invitrogen), and 1 unit of Taq polymerase (Invitrogen). A PTC-200 thermocycler (MJ Research, Waltham, MA, USA) was used, and PCR amplification was applied under the following cycling conditions: an initial denaturation at 95 °C for 10 min followed by 35 cycles. Each cycle included the following steps: a denaturation at 95 °C for 30 s, an annealing at 53 °C for 30 s, and an extension at 72 °C for 1 min. A final extension at 72 °C for 10 min was applied.

The PCR amplification products were separated in 1.5% (wt/vol) agarose gels using 1X Tris Acetate EDTA (TAE) and photographed on a UV transilluminator. PCR amplification products were purified by using the NucleoSpin Extract Kit (Macherey Nagel, Duren, Germany) in order to remove secondary metabolites prior to sequencing. All sequences were determined on an ABI PRISM^®^ 3700 DNA Analyzer (Applied Biosystems). Each fragment used was sequenced in both directions to maximize the accuracy of the sequencing.

### 4.5. Statistical Analyses

Additional sequences from identified Uncinaria species were derived from GenBank database, and Uncinaria stenocephala was inferred as outgroup (Table 4). All datasets were aligned using the Clustal × v2.0 software [108], confirmed by eye, edited, and compiled using the Chromas Pro software, and the extracted sequences were analyzed by using a BLAST search in GenBank in order to verify sequence orthology.

MEGAX software [109] was used to calculate the Disparity Index Test for Heterogeneity to test for homogeneity of substitution patterns between sequences. In order to infer phylogenetic relationships, a main Bayesian inference model-based method was used. MrBayes v.2.01 software was used for Bayesian inference [110]. The dataset was explored by using four chains: one cold chain and subsequently three incrementally heated ones by a temperature set at 0.20. A GTR model of sequence evolution was employed allowing a gamma shape of among site-rate variation. The GTR model normally fits real data better than the other (simpler) alternative models [111]. Through the evolution of speciation, nucleotide substitutions are not at equilibrium; thus, the GTR model predicts this complexity better. Posterior probability distributions were obtained for the phylogenies, and the parameters of the model of sequences’ evolution were adjusted; random trees were used as seeds. Tree spaces were explored inferring 1,000,000 generations, with 100 generations sampled each time, and the burn in was set to 10,000.

## 5. Conclusions

The taxonomic identity of *Uncinaria hamiltoni* and its important role as a significant agent of disease and mortality was reported for the first time in Mediterranean monk seal pups. Hookworm infections can have a detrimental impact on neonatal monk seal pups, considering that nearly half of the studied animals died shortly after entering the rehabilitation program. Its effect on the population is of special concern, as it could become a potential threat and play an important role in population regulation. Even though studying and managing disease in wild marine hosts is complex and challenging, it permits a better understanding and adds valuable knowledge about the health status of specific species and the identification and epidemiology of certain known and novel pathogens.

## Figures and Tables

**Table 1:** Summary of arrival and release/death age, length and weight, and “body mass index” of seal pups that survived and those that died.

	Arrival		Release/Death	
	Survived	Died	Survived	Died
Age	24.9 (3–90)	19.8 (5–65)	143.1 days (104–194)	31.9 days (8–70)
Rehabilitation Time	n/a	n/a	118.3 days (25–174)	12.7 days (0–63)
Length	113.8 cm (105–124)	109.5 (90–123)	136.8 cm (123–145)	111.4 (91–123)
Weight	18.2 kg (13–29.9)	16.3 kg (9–27)	55.4 kg (43.2–60)	16.7 (8–27)
BMI	0.160 (0.111–0.253)	0.148 (0.100–0.225)	0.405 (0.345–0.456)	0.149 (0.141–0.225)
Expected “Healthy” Mean BMI	0.186 (0.162–0.257)	0.180 (0.164–0.230)	0.316 (0.273–0.372)	0.193 (0.167–0.235)
% Difference from “healthy”	−14.1% (−34.5%–15.2%)	−17.0% (−51.6%–26.1%)	29.0% (1.4%–47.4%)	−22.6% (−53.2%–25.3%)
*n*	16	15	16	15

**Table 2:** Hematological and biochemical values of the Monk seal pups at admission to the rehabilitation center.

Hematological and Biochemical Parameters	Range	Minimum	Maximum	Mean	Std. Deviation
Red Blood Cells (RBC) × 1,000,000/μL	3.94	0.78	4.72	2.8	1.08
Hemoglobin (Hgb) gr/dL	126.3	2.7	129	18.42	29.79
Hematocrit (Hct)-Packed Cell Volume (PCV) %	46.7	9.3	55.7	34.3	11.67
Mean corposcular volume (MCV) fl	26	102	128	117.61	8.29
Mean corposcular hemoglobin (MCH) pg	18.3	30.3	48.6	38.33	5.65
Mean corp hemoglobin consentration (MCHC) g/dL	15	25.5	40.5	32.69	4.85
White Blood Cells (WBC) × 1000/μL	18.9	11.7	30.6	21.02	5.41
Neutrophils %	76.8	10.5	87.3	67.62	21.37
Lymphocytes %	72.74	8.76	81.5	28.03	20.45
Monocytes %	20	0	20	5.92	5.35
Eosinophils %	3	0	3	0.82	0.97
Vasophils %	1	0	1	0.2	0.44
Platelets ×1000/μL	665	194	859	571.06	213.22
Total Proteins g/dL	3.9	3.3	7.2	5.71	1.07
Urea mg/dL	137	20.0	157	66.88	46.02
Creatinine mg/dL	1.7	0.2	1.9	0.67	0.38
Aspartate aminotransferase (AST) U/L	1001	38	1039	162.94	234.99
Alanine transaminase (ALT) U/L	319	19	338	71.05	81.79
Alkaline phosphatase (ALP) U/L	123	34	157	71	39.66
Lactate dehydrogenase (LDH U/L	4134.8	811	4945.8	1974.75	1377.14
Calcium mg/dL	2	8.6	10.6	9.58	0.65
Phosphorus mg/dL	1.7	5.7	7.4	6.68	0.55
Cholesterole mg/dL	2186	119	2305	448.35	751.44
Triglycerides mg/dL	82	37	119	69.175	28.9
Albumins g/dL	3.2	0.4	3.6	2.4	1.19
Glucose mg/dL	162	5	167	69.9	46.94
Potassium mmol/L	1.79	4.21	6	5.1	0.6
Sodium mmol/L	13.8	136.3	150.1	145.7	4.78

**Table 3:** Hookworm intensity, body condition, and hematocrit of stranded Mediterranean monk seal pups (*n* = 31).

Number of Mediterranean Monk Seal Pups	Body Condition	Mean Number of Hookworm Eggs/10 Micr Fields	Hematocrit (PCV)	Outcome
5	Critical x < −35%	112	9–18%	5 Died
11	Poor 15% < x < −35%	24	18–28%	3 Died 8 Survived
15	Good/Fair x > −15%	10	28–55.7%	7 Died 8 Survived

**Table 4:** NCBI genomic regions for four available identified *Uncinaria* species and taxa used for phylogenetic analyses. Accession numbers in bold were recently submitted to GenBank as an outcome of the present study. *U. stenocephala* sequences were used as an outgroup. N/A: not available sequences in NCBI database.

Species	COI	ITS	D2/D3 28S
*U. lucasi*	MT154514.1 [31] MT154516.1 [31]	HQ262154.1 [30] HQ262153.1 [30]	HQ261827.1 [30] HQ261823.1 [30]
*U. hamiltoni*	MW581887 (this study)	HE962184.1 [30] HE962183.1 [30] MW581839 (this study)	HQ261869.1 [30] HQ261867.1 [30] MW581843 (this study)
*U. sanguinis*	KF693746.1 [32] KF693748.1 [32]	KF690669.1 [33] KF690668.1 [33]	N/A

**Table 5:** Mean PCV’s of seal pups, egg counts found in feces, and treatment applied in survived and deceased Mediterranean monk seal pups during the rehabilitation period.

	Day 0 (Arrival)		Day 15		Day 30		Day 45		Day 60	
	Survived	Died during 1st week)	Survived	Died	Survived	Died	Survived	Died	Survived	Died
Mean PCV % No of pups	35.3 (*n* = 16)	31.1 (*n* = 15)	37.9 (*n* = 16)	0	40.1 (*n* = 16)	0	41.9 (*n* = 16)	0	42.9 (*n* = 16)	0
Egg count (mean per 10 optical field)	17.0	47.0	5.0	NA	0	NA	0	NA	0	NA
Drug given (oral)	fenbendazole 10 mg/kg b.w. × 3 days or mebendazole 20 mg/kg b.w. × 2 days and amoxycillin+clavulanic 12 mg/kg b.w. or metronidazole 10 mg/kg b.w. × 10 days		fenbendazole 10 mg/kg b.w. or mebendazole 20 mg/kg b.w.		fenbendazole 10 mg/kg b.w. or mebendazole 20 mg/kg b.w.		No treatment		No treatment	

## Data Availability

Not applicable.
